# A national study of choanal atresia in tertiary care centers in Canada – part II: clinical management

**DOI:** 10.1186/s40463-021-00503-3

**Published:** 2021-07-13

**Authors:** Josee Paradis, Agnieszka Dzioba, Hamdy El-Hakim, Paul Hong, Frederick K. Kozak, Lily H. P. Nguyen, Demitri Perera, Evan Jon Propst, Jennifer M. Siu, Monika Wojtera, Murad Husein, National Choanal Atresia Study Working Group

**Affiliations:** 1 Department of Paediatric Otolaryngology- Head and Neck Surgery, Children’s Hospital at London Health Sciences Centre, London, ON Canada; 2 Otolaryngology-Head and Neck Surgery, Schulich School of Medicine and Dentistry, Western University, London, ON Canada; 3 Division of Pediatric Surgery and Otolaryngology Head and Neck Surgery, Departments of Surgery and Pediatrics, The Stollery Children’s Hospital, University of Alberta Hospital, Edmonton, AB Canada; 4 IWK Health Centre, Halifax, NS Canada; 5 Division of Otolaryngology-Head and Neck Surgery, Department of Surgery, Dalhousie University, Halifax, NS Canada; 6 Faculty of Medicine, University of British Columbia, Vancouver, BC Canada; 7 Division of Pediatric Otolaryngology-Head and Neck Surgery, BC Children’s Hospital, Vancouver, BC Canada; 8 Department of Otolaryngology – Head and Neck Surgery, McGill University, Montreal, Canada; 9 Institute for Health Science Education, McGill University, Montreal, Canada; 10 Department of Pediatric Surgery, Montreal Children’s Hospital, Montreal, Canada; 11 Faculty of Medicine, University of Queensland, Brisbane, Queensland Australia; 12 Department of Otolaryngology–Head & Neck Surgery, Hospital for Sick Children, University of Toronto, Toronto, ON Canada

**Keywords:** Choanal atresia, Surgical repair, Post-operative management, Planned second look, Surgical adjuncts

## Abstract

**Background:**

To evaluate the clinical management of choanal atresia (CA) in tertiary centers across Canada.

**Methods:**

Multi-centre case series involving six tertiary care pediatric hospitals across Canada. Retrospective chart review of patients born between 1980 and 2010 diagnosed with choanal atresia to a participating center.

**Results:**

The health charts of 215 patients (59.6% female) with choanal atresia (CA) were reviewed. Mean age of initial surgical repair was 0.8 months for bilateral CA, and 48.6 months for unilateral CA. Approaches of surgical repair consisted of endoscopic transnasal (31.7%), non-endoscopic transnasal (42.6%), and transpalatal (25.2%). Stents were used on 70.7% of patients. Forty-nine percent of patients were brought back to the OR for a planned second look; stent removal being the most common reason (86.4%). Surgical success rate of initial surgeries was 54.1%. Surgical technique was not associated with rate of restenosis [χ^2^ (2) = 1.6, *p* = .46].

**Conclusions:**

The present study is the first national multi-institutional study exploring the surgical outcomes of CA over a 30-year period. The surgical repair of CA presents a challenge to otolaryngologists, as the rate of surgical failure is high. The optimal surgical approach, age at surgical repair, use of stents, surgical adjuncts, and need for planned second look warrant further investigation.

**Graphical abstract:**

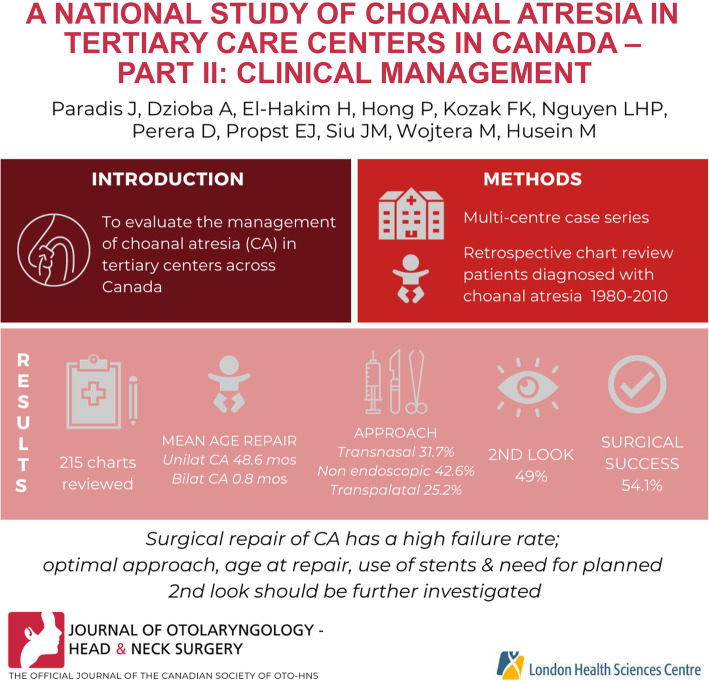

## Background

Choanal atresia (CA) is a congenital condition resulting in obstruction of the posterior nasal passage(s), known as the choana, with an incidence of approximately one in 5000 to 8000 live births [1, 2]. Management of CA involves surgical resection of the atretic plate and surrounding structures [3]. The most common approaches to surgical repair of CA include endoscopic transnasal (ETN), non-endoscopic transnasal (NTN), and transpalatal (TP), with the ETN being the currently favored approach [4]. In addition, adjunct procedures are often employed including laser-assisted surgery, placement of nasal stents, and use of anti-proliferatives [4–8].

To date, best practices for management of CA remain to be determined. Method of surgical correction of CA is usually dictated by surgeon or institutional preference [9]. Postoperative, rates of restenosis remain unclear [7, 9]. Controversies regarding the use of stents in aiding surgical correction of CA [4, 5], minimizing restenosis as well as the use of other adjunct procedures such as laser [6, 7] and Mitomycin-C [8] remain unresolved. Furthermore, the purpose and benefit of following up in the operating room with a planned second look post initial surgical repair is under-reported in the literature.

The reasons for these unresolved controversies regarding best practices for clinical management of CA is partly due to the rarity of the disorder and consequently the limited level of evidence available in the literature. Published studies often report case series of single surgeon or single institution experiences, involving small sample sizes [9–11], with few studies reporting treatment outcomes on samples sizes larger than 30 [11–16].

The aim of the current study is to elucidate controversies surrounding the management of CA using a multi-institutional approach. The Canadian landscape lends a unique setting to evaluate the management of CA, as all CA repairs are performed in academic settings. In a two-part study, this national investigation provides a comprehensive review of the clinical presentation (Part I) and management (Part II) of a large sample of patients with CA treated at tertiary care centres across Canada. The present paper reports on the clinical management of CA. Specifically, the following outcomes were explored: surgical management and use of adjunct procedures, utilization of a post-operative planned second look, and success rate of surgical repair for CA.

## Methods

Pediatric otolaryngologists practicing in tertiary care centers across Canada were invited to take part in the study via phone or email. Of the nine healthcare centers who were contacted, six centers (Western University in London, University of Toronto in Toronto, University of Alberta in Edmonton, University of British Columbia in Vancouver, McGill University in Montreal and Dalhousie University in Halifax) agreed to participate in the chart review. Health records of patients born between 1980 and 2010 diagnosed with CA who underwent treatment at participating centers were included in this study. Patients born before 1980 or after 2010 or patients who did not receive a definitive diagnosis of CA were excluded from the study. A standardized checklist was completed for each patient presenting with CA who met study inclusion/exclusion criteria. The following variables were collected from patient chart review: surgical approach, surveillance, surgical instruments, adjuncts to surgery, findings of planned second look, revision surgeries, reasons for revision surgeries, and, a comparison of surgical revision rates between surgical approaches, and for bilateral versus unilateral cases of CA. In the current sample, surgical success was defined as presence of choanal patency, with no need for surgery for restenosis or debridement of granulation tissue after initial repair. Each participating center obtained ethical approval for this study from their institution’s Health Research Ethics Board.

### Data analysis

A descriptive analysis of study outcomes is presented. In addition to descriptive statistics, results of chi-square tests/Fisher’s exact tests, independent samples t-tests, and Spearman rho correlation coefficients were reported where appropriate. Statistical tests were conducted with SPSS (IBM Corp. Released 2017. IBM SPSS Statistics for Windows, Version 25.0. Armonk, NY: IBM Corp). Alpha was set a priori at .05 to determine statistical significance.

## Results

Two-hundred and fifteen patients across the six participating centers [London (*n* = 26), Toronto (*n* = 83), Edmonton (*n* = 17), Vancouver (*n* = 60), Montreal (*n* = 11), Halifax (*n* = 18)] met the study inclusion/exclusion criteria and were included in this national study. Health records of 215 patients were reviewed. One-hundred and twenty-seven patients (59.6%) were female and 88 (40.9%) were male. The mean age of patients at time of CA presentation was 0.4 months (range 0.1 to 7.2 months) for bilateral CA and 37.8 months (range 0.1 to 164.1 months) for unilateral cases.

### Surgical repair

Three common surgical techniques were utilized on patients for correction of CA in the Canadian tertiary care centers: endoscopic transnasal (ETN) (31.7%), non-endoscopic transnasal (NTN) (42.6%) and transpalatal (TP) (25.2%) techniques. The ETN technique was used from 1992 to 2010; NTN was used from 1980 to 2010; and, the TP technique was used from 1985 to 2010. Table 1 displays information on the surgical correction and surveillance of this patient cohort. The mean age at initial surgical repair for CA was 28.5 months: 0.8 months for bilateral CA and 48.6 months for unilateral CA. For patients who underwent ETN surgery, common surgical instruments included the stammberger punch, urethral sound dilators, suction punch and backbiting forceps. Adjuncts to the ETN technique included two reports (3.1%) of Mitomycin-C use and two reports (3.1%) of Holmium-YAG laser assistance. For patients who underwent the NTN surgery, commonly reported surgical instruments used included the Skeeter drill, Stammberger punch, urethral sound dilation, suction punch and backbiting forceps. Adjuncts to the NTN approach were more frequently reported than the ETN and TP approaches and included 33 cases (34.4%) of laser use (30 carbon dioxide and 3 Holmium-YAG) and 8 cases (9.3%) of Mitomycin-C use. Finally, for patients who underwent the TP surgery, common surgical instruments included drills, suction punch, bone punch and bone rongeurs. There was only one reported case (2.0%) of Mitomycin-C use and no laser use with the TP surgical technique subgroup.

**Table 1:** Surgical Repair and Surveillance of Choanal Atresia (*n* = 215)

Variable	Category	No. (%)
Age at repair (months)	mean (SD)	28.54 (43.0)
Type of surgery	Transnasal (endoscopic)	64 (31.7)
	Transnasal (non-endoscopic)	86 (42.6)
	Transpalatal	51 (25.2)
	Other	1 (0.5)
Nasal Stents	Yes	128 (70.7)
	No	53 (29.3)
Stent duration (days)	mean (SD)	39.6 (50.7)
	range	2.1 to 270.0
Planned second look	Yes	81 (49.1)
	No	84 (50.9)

Stents were used in 70.7% of patients (*n* = 128) and almost exclusively involved the use of endotracheal tubes that varied in size from 3 to 5.5 mm; the exception was one placement of an 18 French latex urological catheter and one use of the nasal trumpet as a stent. Stents were placed for an average of 39 days. Forty-nine percent (*n* = 81) of patients were brought into the OR after their initial repair for a planned second look. Reasons for a planned second look included stent removal or replacement (86.4%), debridement of tissue granulation (17.3%), examination with nasendoscopy, laryngoscopy or bronchoscopy (8.6%), laser revision (1.2%), and clot formation under the hard palate (1.2%). Patients who had nasal stents inserted were statistically significantly more likely to require a planned second look in the OR than patients who did not have nasal stents (64.0 vs 14.0%) [χ^2^ (1) = 34.8, *p* < .001].

Patients with bilateral CA were significantly more likely to have stents placed compared to patients with unilateral CA [(68.5% vs 53.7%), χ^2^(1) = 17.7, *p* < .001]. Patients with bilateral CA also had their stents inserted for an average of 1 month longer than patients with unilateral CA [*t* (211) = − 4.41, *p* < .001].

Findings of the planned second look indicated a patent choana (16.0%), granulation tissue (16.0%), restenosis (4.9%), and mucus crust plugging of nasopharynx (2.5%), with most case reviewed (60.5%) not reporting findings of the planned second look.

### Revision surgeries

Table 2 displays information on revision rates and surgical techniques. One hundred and five patients (50.7%) underwent a 1st revision surgery. The mean (SD) time from initial surgical repair to 1st revision surgery was 18.2 (30.6) months. Reasons for revision surgery included restenosis (73.3%), debridement of granulation tissue (11.4%), stent replacement, repositioning or removal (9.5%) and unknown (5.7%). As such, of the 207 patients with known information on revision surgery, 112 patients did not require a revision surgery or underwent a revision surgery solely for the purpose of stent removal, repositioning, or replacement, resulting in a surgical success rate of 54.1%. In contrast, 95 patients required 1 or more revision surgeries for restenosis or presence of granulation tissue, representing a surgical failure rate of 45.9%. Adjuncts to the 1st revision surgery included 32 cases (30.5%) of laser use (30 CO2 and 2 Holmium-YAG) and 4 cases (3.8%) of Mitomycin-C use.

**Table 2:** Revision Surgeries

Variable	Category	1st Revision No. (%)	2nd Revision No. (%)	3rd Revision No. (%)
Revision required?	Yes	105 (50.7)	51 (25.6)	26 (13.5)
	No	102 (49.3)	148 (74.4)	166 (86.5)
Post-op time at revision (months)	mean (SD)	18.2 (30.6)	40.6 (48.0)	43.4 (48.1)
Type of Revision	ETN	30/105 (28.6)	14/51 (27.5)	9/26 (34.6)
	NTN	59/105 (56.2)	29/51 (56.9)	14/26 (53.8)
	TP	12/105 (11.4)	5/51 (9.8)	0/26 (0)
	Unknown	4/105 (3.8)	3/51 (5.9)	3/26 (11.5)

*Note*. *ETN* Endoscopic transnasal, *NTN* Non-endoscopic transnasal, *TP* Transpalatal

Fifty-one patients (25.6%) underwent a 2nd revision surgery. The mean (SD) time from initial surgical repair to 2nd revision surgery was 40.6 (48.1) months. Reasons for 2nd revision included: restenosis (80.4%), debridement of granulation tissue (3.9%), stent removal (9.8%), and unknown (5.9%). Adjuncts to 2nd revision surgeries included 22 cases (43.1%) of laser use (19 CO2 and 3 Holmium-YAG) and 3 cases (5.9%) of Mitomycin-C use. Twenty-six patients (12.1%) underwent a 3rd revision surgery. The mean (SD) time from initial surgical repair to 3rd revision surgery was 43.4 (48.1) months. Reasons for a 3rd revision included: Restenosis (69.2%), debridement of granulation tissue (11.5%) and stent removal (19.2%). Adjuncts to the 3rd repair surgeries included 11 cases (42.3%) of laser use (10 CO2 and 1 Holmium-YAG) and 1 case (3.8%) of Mitomycin-C use. Table 3 displays rates of adjunct procedures for primary and revision surgeries.

**Table 3:** Rates of Adjunct Procedures for Primary and Revision Surgeries for CA

Surgery	Laser No. (%) Patients	Mitomycin-C No. (%) Restenosis
Primary Surgery (n = 215)	35 (16.3%)	11 (5.1%)
1st Revision (*n* = 105)	32 (30.5%)	4 (3.8%)
2nd Revision (*n* = 51)	22 (43.1%)	3 (5.9%)
3rd Revision (*n* = 26)	11 (42.3%)	1 (3.8%)

The median number of revision surgeries was 1 with a range of 0 to 8 revision surgeries for the study cohort. Table 4 displays rates of restenosis by initial surgical approach. Similar restenosis rates were found for all three surgical approaches (ETN, NTN, TP). Surgical technique was not statistically significantly associated with rate of restenosis [χ^2^ (2) = 1.6, *p* = .46].

**Table 4:** Rate of Restenosis by Initial Surgical Repair Type

Surgical Technique	No. (%) Patients	No. (%) Restenosis
Transnasal (endoscopic)	64 (31.8)	23 (35.9)
Transnasal (non-endoscopic)	86 (42.8)	36 (41.9)
Transpalatal	51 (25.4)	18 (35.3)

### Bilateral versus unilateral CA

Table 5 displays surgical revision rates, comparing bilateral versus unilateral CA. Patients with bilateral CA were statistically significantly more likely to have stents placed compared to patients with unilateral CA [(68.5% vs 53.7%), χ^2^(1) = 17.7, *p* < .001]. Patients with bilateral CA also had their stents inserted for an average of 1 month longer than patients with unilateral CA [*t* (211) = − 4.4, *p* < .001].

**Table 5:** Comparison of Bilateral versus Unilateral Choanal Atresia

Variable	Unilateral (***n*** = 122) No. (%)	Bilateral (***n*** = 93) No. (%)	***p***-value
Stents	65 (53.7)	63 (68.5)	<.001
Mean (SD) stent duration (days)	27 (36)	56.7 (63.0)	<.001
1st Revision Surgery	52 (44.4)	53 (58.9)	.10
2nd Revision Surgery	20 (17.9)	31 (35.6)	.004
3rd Revision Surgery	5 (4.7)	21 (24.7)	<.001

*Note*. *p*-values represent results of an independent samples t-test for stent duration variable and results of chi-square tests for the remaining variables

Patients with bilateral CA were not significantly more likely to require a 1st revision surgery than unilateral CA [(58.9% versus 44.4%), χ^2^(1) = 2.8, *p* = .10]. However, patients with bilateral CA were significantly more likely to require a 2nd and 3rd revision surgery [χ^2^(1) = 8.4, *p* = .004; χ^2^(1) = 16.5, *p* < .001, respectively] than patients with unilateral CA. Individuals with unilateral CA had a median of 1 revision surgery (range 0 to 3 revision surgeries), while individuals with bilateral CA had a median of 2 revision surgeries (range 0 to 8 revision surgeries).

## Discussion

Choanal atresia presents a surgical challenge to otolaryngologists as the atresia often involves multiple structures including the nasal septum, atretic plate, lateral wall and skull base [17], and has a high rate of restenosis and need for revision surgery. The present investigation explored the clinical management and surgical outcomes of CA over a 30-year period (1980 to 2010). This is the first national multi-institutional study reporting on a large sample size of 215 patients with CA. This study will contribute to the body of knowledge on CA, particularly relative to surgical correction of the condition.

### Surgical repair

Surgical success rates for CA repair vary widely in the literature and range from 0 to 85% [17–20]. The large range in outcome is likely attributed to variability in surgical technique, study sample, sample sizes, and definition of surgical success, which is not operationally defined in most published works or standardized across studies [21]. We observed a surgical success rate of 54.1% for the entire cohort with the primary surgery. Eladl and Khafagy (2016) reviewed 112 cases of bilateral CA using transnasal endoscopic CA repair and reported a 42% restenosis rate [17], suggesting a 58% success rate. The present study also found no difference in rates of 1st revision surgeries between the unilateral and bilateral cases of CA, but statistically significantly higher rates of 2nd (*p* = .004) and 3rd (*p* < .001) revision surgeries for bilateral CA. Similarly, Kinis et al. (2014) reported on the success rate of the ETN approach on 33 patients with CA and reported a 53.8% restenosis rate for bilateral CA and 23.1% restenosis rate for unilateral CA [19].

The present investigation found no statistically significant differences in rates of restenosis between the three surgical repair approaches (ETN, NTN, TP). To the authors’ knowledge, no other studies to date have statistically compared differences in rates of restenosis across different surgical techniques. ETN seems to be the favored approach for CA repair reported in the literature [22], although in our sample, the NTN approach was used most often. However, considerable advances in endoscopic visualization, surgical techniques, stents, have occurred over the 30-year study period. Other authors have reported that the ETN repair is safe and effective, resulting in good outcomes with or without stenting [4]. Various studies have reported surgical success rates for the ETN approach that range from 67 to 88% [5, 10], while a meta-analysis of 20 studies reported a mean success rate of 85.3% for ETN approach [23]. The TP approach is often indicated in cases of exceptionally thick bony atresia or nasal alar stenosis [12]. However, due to high changes of cross-bite and high arch deformity, the TP repair is not recommended for children younger than 6 years [21]. As such, the optimal surgical approach for CA repair remains unclear and is likely dictated by the anatomical involvement of the atresia, technological advances and surgeon preference.

### Adjunct procedures

The need and utilization of a planned second look in the OR to evaluate surgical outcome following initial atresia repair is under-reported in the literature. A planned second look was undertaken in 49.1% of the current study cohort. Patients who had nasal stents inserted were statistically significantly more likely to require a planned second look in the OR (64.0%) than patients who did not have nasal stents (14.0%) (*p* < .001) in the current study cohort. In Eladl & Khafagy’s review of 112 cases of bilateral CA, they reported a higher rate of second-look procedures than the present study, where 61% of cases had undergone the procedure. However, consistent with the present study, Eladl & Khafagy’s study also found that the need for a second-look evaluation was largely attributed to stenting in that 74.5% of patients with a stent compared to 20.6% who did not have stent placement had undergone a second-look evaluation [17]. As such, nasal stent repositioning, replacement or removal was the primary motive for a planned second look. The benefit of a planned second look for non-stent cases require further investigation. For example, Eladl & Khafagy [17] suggest that post-operative dilation using dilators under endoscopic examination after initial repair of bilateral CA helps prevent restenosis and need for planned second look. Further investigation into the utilization and benefit of a planned second look following CA repair and the use of imaging to assess for recurrence is warranted.

To date, the value of adjuvant procedures such as Mitomycin-C, laser assisted techniques, and stenting is not proven [9, 12, 24]. Newman et al. (2013) found no statistically significant difference in the rate of restenosis for patients treated with Mitomycin-C, stenting, or subsequent dilation in 39 cases of CA in those treated with the ETN approach [12], while Bozkurt et al. (2010) found that the rate of restenosis was lower in 6 patients treated with Mitomycin compared with 14 CA patients not treated with Mitomycin following ETN repair [25]. In the present investigation, laser assisted surgery occurred most often with the NTN approach, wherein 38.4% of surgeries (*n* = 33) were assisted with carbon dioxide or holmium-YAG lasers, while 3.1% of ETN and 0% of TP surgeries utilized lasers to correct CA. Mitomycin-C was used infrequently as an adjunct to surgical repair and again was used more often following NTN approaches (9.3%), than ETN (3.1%) or TP (2.0%) approaches. Mitomycin-C is no longer used as an adjunct to surgical repair due to its carcinogenic properties.

Post-operative stenting has not been proven to increase chances of surgical success [5, 21], even though authors still recommend placement of stents in high risk cases such as in neonates and bilateral CA [24]. A recently published meta-analysis of 15 studies evaluating bilateral CA repair found similar surgical success rates for stented (65%) versus non-stented (64%) patients [5]. Stent duration varies across studies from a couple of days to a few months [5]. In the present investigation, for the 70.7% of patients who were stented, stents were placed for an average of 1.3 months, with patients with bilateral CA having stents inserted for a month longer, on average, than unilateral cases.

### Study limitations

Although this large multi-center study presents a significant addition to the CA literature, several study limitations should be noted. First, due to the retrospective nature of this study, patients were not randomized to intervention groups and this study found significant variability in the instrumentation used, and adjunct procedures used across treatment groups. As such, rates of restenosis across the three surgical approaches (ETN, NTN, TP) may be confounded by patient characteristics [bilateral vs. unilateral CA, anomalies, nature of atresia (bony, membranous, mixed), thickness of the atresia, etc.] and use of adjunct procedures. As this was a retrospective study, these patient and treatment factors could not be accounted for. To date, only two small randomized controlled trials have been conducted on CA; one evaluating Mitomycin-C use versus no-use in 20 children who were treated with the ETN approach [26] and one comparing stenting versus no stenting in 20 children with bilateral CA also treated with the ETN approach [27], while RCTs comparing different surgical techniques have not been conducted to date. Prospective RCTs comparing treatment approaches, stenting vs. no stenting, Mitomycin-C, laser assisted surgery, and type of atresia would help further clarify these outstanding controversies around the management of CA.

Furthermore, three tertiary care centres in Canada did not participate in the present investigation, limiting the generalizability of study findings. Finally, given the multi-institutional nature of this study, results of the study may be confounded by institutional factors including variability in surgeon expertise, institutional preferences for use of different surgical techniques, time of repair and use of adjunct procedures. This variability across sites may limit the interpretability of study findings.

## Conclusions

This retrospective multi-center investigation reported on over 30-years experience of patients with CA across tertiary care centers in Canada. Surgical repair type, whether it be ETN, NTN, or TP was not statistically significantly associated with rate of restenosis, while a weak negative association between age and number of revision surgeries was found. Stenting was used often in this patient cohort and stent removal, repositioning, and replacement were the primary reasons for a planned second look in the OR following initial surgical repair. Laser use facilitated NTN approaches most frequently, with limited use in ETN and no use in TP approaches. Mitomycin-C was used infrequently in the present patient cohort. Future investigations regarding hereditary linkages, the need for a planned second look, stenting, laser and Mitomycin-C use, and surgical repair approaches are warranted.

## Data Availability

The datasets used and/or analyzed during the current study are available from the corresponding author on reasonable request.

## Abbreviations

CA: Choanal atresia
ETN: Endoscopic transnasal
NTN: Non-endoscopic transnasal
TP: Transpalatal
